# Ceftriaxone-induced cholestatic hepatitis in a child: A case report and a review of the literature

**DOI:** 10.3389/fped.2022.1051887

**Published:** 2022-12-05

**Authors:** Massimo Luca Castellazzi, Carlo Virginio Agostoni, Jessica Palella, Daniela Civeriati, Paola Marchisio, Gabriella Nebbia

**Affiliations:** ^1^ Pediatric Emergency Department, Fondazione IRCCS Ca’ Granda Ospedale Maggiore Policlinico, Milan, Italy; ^2^ Pediatric Intermediate Care Unit, Fondazione IRCCS Ca’ Granda Ospedale Maggiore Policlinico, Milan, Italy; ^3^ Department of Clinical Sciences and Community Health, University of Milan, Milan, Italy; ^4^ University of Milan, Milan, Italy; ^5^ Pediatric Highly Intensive Care Unit, Fondazione IRCCS Ca’ Granda Ospedale Maggiore Policlinico, Milan, Italy; ^6^ Department of Pathophysiology and Transplantation, University of Milan, Milan, Italy

**Keywords:** ceftriaxone, hepatitis, cholestasis, children, antibiotic

## Abstract

Ceftriaxone, a third-generation cephalosporin, is commonly used in pediatric patients and is generally well tolerated. Its more frequent adverse effects are biliary pseudolithiasis, urolithiasis, and hemolytic anemia. On the other hand, ceftriaxone-induced acute cholestatic hepatitis is a very rare condition, especially in children. Here, we describe a case of this condition in a young male child to highlight the importance of suspecting this drug-induced liver injury to achieve a prompt diagnosis.

## Introduction

Ceftriaxone is a third-generation cephalosporin with broad-spectrum antimicrobial activity and is widely used in pediatric patients of all ages (1).

It is excreted mainly by the kidneys, whereas nearly 40% is excreted through the bile without being metabolized (2).

Generally, ceftriaxone is well tolerated. Possible adverse drug reactions of ceftriaxone include gastrointestinal disorders such as nausea, vomiting, and diarrhea, biliary pseudolithiasis, urolithiasis, and hemolytic anemia (1).

Ceftriaxone-induced acute hepatitis is a rare condition described in pediatric patients (3, 4).

Here, we describe one of these cases to highlight the importance of suspecting this condition to achieve a prompt diagnosis.

## Case presentation

A previously healthy 5-year-old boy was admitted to our pediatric emergency department with a history of low-grade fever (maximum axillary temperature of 38 °C) and left later cervical painful swelling since the day before. For fever, the patient was treated with paracetamol at an appropriate dosage based on his weight. The patient denied vomiting or diarrhea and had no direct contact with sick people, recent travels, or blood transfusion. No history of allergy was reported, and, in particular, no drug allergies were known.

On admission, the patient was in good general condition, apyretic, and with normal vital signs. His pharynx was only slightly hyperemic. In the later cervical region, there was a swelling of about 3 × 2 cm in size, with hard elastic consistency, mobile, painful on palpation, and normal overlying skin. The results of the cardiorespiratory examination were normal. The liver and the spleen were nonpalpable. No skin rash was observed.

The patient was tested for SARS-CoV-2 with polymerase chain reaction molecular testing on a nasopharyngeal swab, which resulted in negative. Moreover, there was no history of close contact with persons with SARS-CoV-2 infection.

Laboratory tests performed on admission showed the following: C-reactive protein (CRP) 1.9 mg/dl (normal value <0.5), white blood cell count 9,150/mm^3^ with 68.8% neutrophils, and 21.9% lymphocytes. Liver function tests were normal, without any signs of cholestasis. Table 1 shows the patient's blood test results in detail. Furthermore, an ultrasound of the neck was performed, showing enlarged oval lymph nodes, with the largest being 3 × 1.2 cm in diameter on the left side and with a modest increase of vascularization at the echocolordoppler. A blood culture resulted negative. Broad-spectrum intravenous antibiotic treatment with ceftriaxone (75 mg/kg/day) was started with rapid clinical improvement of the later cervical lymphadenitis.

**Table 1:** Laboratory data of the 5-year-old child with ceftriaxone-induced acute cholestatic hepatitis.

Laboratory data (normal value)	T0 days	T3 days	T4 days	T5 days	T7 days	T10 days	T20 days	T40 days
WBC, cells/ml (4,800–12,100)	9,150	6,240	8,040	6,270	11,290	7,710	4,510	4,380
Neutrophils, %	68.8	82.2	66.9	66.2	70	59.1	51.6	35
Eosinophils, %	1.2	2.9	2.5	2.7	1	2	3	2.5
Hemoglobin, g/dl (10.5–14.5)	13.3	12.6	12.5	12	11,7	12	12.1	12.3
CRP, mg/dl (<0.06)	1.9	2.04	1.05	0.52	0.43	0.09	0.07	0.06
AST, U/L (20–55)	18	688	185	67	49	58	49	30
ALT, U/L (10–30)	15	700	494	301	185	118	76	19
Gamma-GT, U/L (5–33)	10	142	141	111	99	81	59	13
Total bilirubin mg/dl (0.12–1.10)	0.41	2.37	2.31	1.88	3.35	0.94	0.65	0.38
Direct bilirubin, mg/dl (0.00–0.30)	NA	1.95	2.23	1.86	3.41	NA	NA	NA
Indirect bilirubin, mg/dl (0.00–0.80)	NA	0.42	0.08	0.02	0.04	NA	NA	NA
Alkaline phosphatase, U/L (142–335)	NP	NP	NP	406	465	393	305	235
INR (0.84–1.20)	NP	NP	1.18	NP	1.17	NP	NP	1.15
aPTT ratio (0.86–1.20)	NP	NP	1.03	NP	1.15	NP	NP	1.05
EBV-DNA	Negative	NP	Negative	NP	NP	NP	Negative	Negative

WBC, white blood cell; CRP, C-reactive protein; AST, aspartate aminotransferase; ALT, alanine aminotransferase; Gamma-GT, gamma-glutamyl transferase; INR, international normalized ratio; aPTT ratio, activated partial thromboplastin time ratio; NP, not performed; NA, not applicable.

Serologic tests for varicella virus, human herpes virus type 1-2-6-8, coxsackie virus, adenovirus, cytomegalovirus, *Toxoplasma gondii*, *Mycoplasma pneumoniae*, and *Borrelia burgdorferi* resulted negative, as well as the antistreptolysin title and oropharyngeal swab. Epstein–Barr virus (EBV) infection was serologically detected, with anti-VCA IgM positivity and the absence of anti-VCA IgG, anti-EA, and anti-EBNA. Nevertheless, EBV-DNA was persistently undetectable. On day 3, the patient reported fatigue and asthenia. He also developed mild scleral jaundice and dark urine. Laboratory tests were performed, showing acute cholestatic hepatitis with an increase of serum transaminase levels, with aspartate aminotransferase (AST) and alanine aminotransferase (ALT) levels higher than 12 and 20 times, respectively, the upper limits of normality (ULNs). Furthermore, total bilirubin was 2.4 mg/dl (normal value <1.1), with direct bilirubin of 1.95 mg/dl, and gamma-glutamyl transferase (gamma-GT) was 142 U/L (normal value <33). Hemoglobin levels were always optimal, and there was no increase in hemolysis indices. Furthermore, the eosinophil count was within the normal range. To exclude other causes of acute hepatitis, serologic tests for B, C, and E hepatitis were performed, resulting negative, as well as antinuclear antibodies (ANAs). An ultrasound examination showed a minimal enlarged liver size with normal parenchyma. The gallbladder was normal, and intra- and extrahepatic biliary tracts were not dilated. Based on these findings, a diagnosis of ceftriaxone-induced hepatitis was performed, and the antibiotic was therefore immediately suspended without replacing it with other antibiotics. As the patient was apyretic since the day of admission, no other drugs were administered. In a few days, a significant improvement of biochemical data was documented, and on day 10, values of AST 58 U/L (normal value <55 U/L), ALT 118 U/L (normal value <33 U/L), gamma-GT 81 U/L, total bilirubin 0.94 mg/dl were documented. Considering this evolution, a liver biopsy was unnecessary. After 40 days, all the liver function tests were below the upper limit of normality.

**Figure 1:**
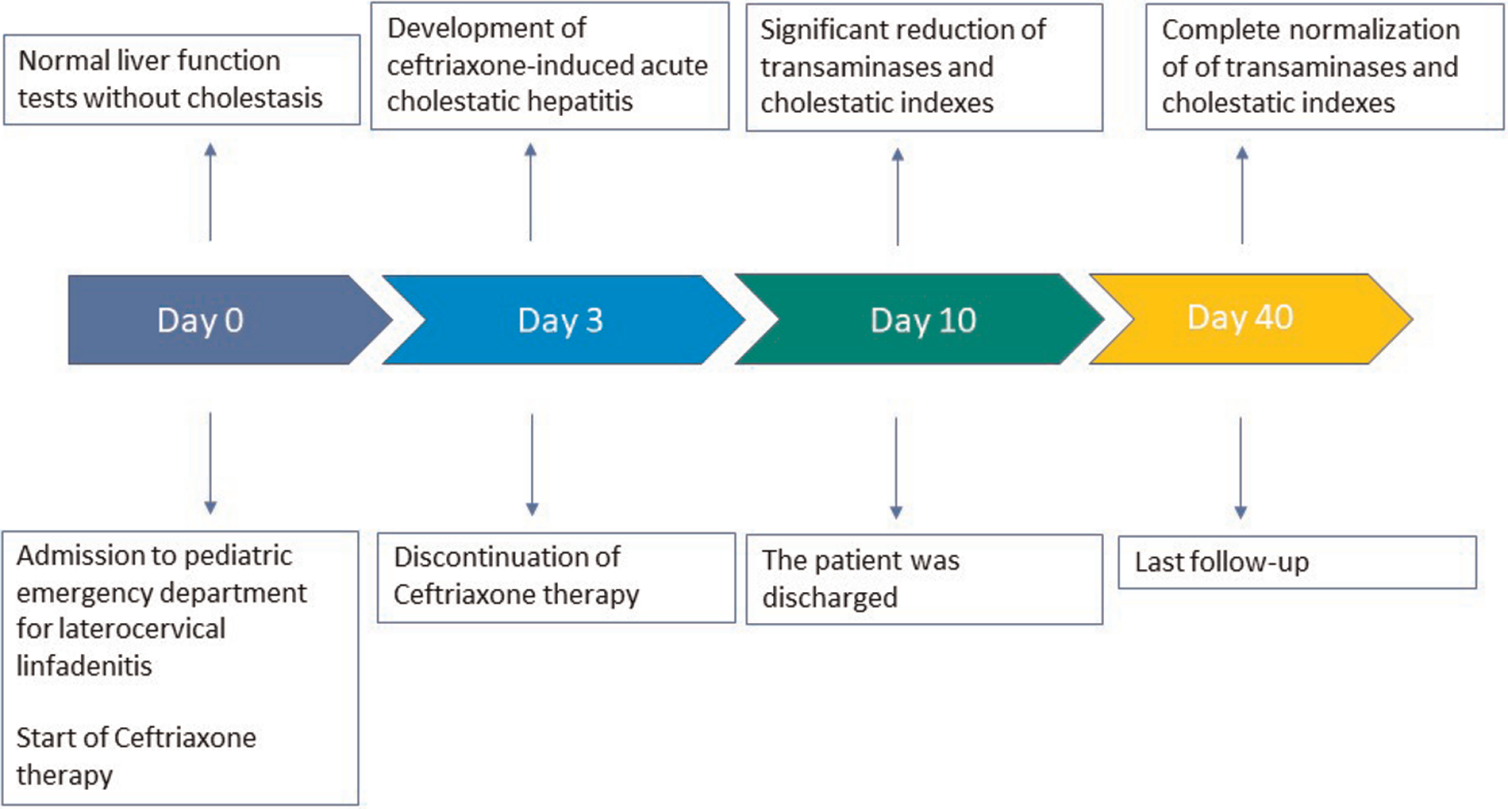
Timeline course of the patient.

A timeline of the clinical course and the laboratory tests is summarized in Figure 1.

## Discussion

Acute cholestatic hepatitis associated with acute EBV infection is rarely described in pediatric age. In a recent review by Shkalim-Zemer et al., 17 cases of this condition were reported. They developed jaundice or icteric sclerae (94%), followed by fever (94%), cervical or generalized lymphadenopathy (73%), and splenomegaly (53%). Only one patient developed a fulminant hepatic failure and died, whereas all the other patients fully recovered without sequelae (4).

In our patient, a serologically acute EBV infection was documented, but EBV-DNA was persistently undetectable.

In the reported case, the direct correlation between the use of ceftriaxone and the development of acute cholestatic hepatitis was supported by the fact that the liver function tests performed before starting the antibiotic therapy were normal and then rapidly improved after its discontinuation. Furthermore, a prominent increase in the levels of gamma-GT suggests a toxic cause (5). Other possible causes of infectious and autoimmune hepatitis were excluded, supporting our diagnostic hypothesis. A possible final confirmation of the diagnosis by liver biopsy was not considered ethically correct.

Furthermore, the Roussel Uclaf Causality Assessment Method (RUCAM) is a scoring system useful in determining the likelihood that a specific treatment causes hepatic injury (6, 7).

It analyzes six factors and assigns a specific score to any of these factors: (1) time to onset, (2) course, (3) risk factors, (4) concomitant drugs, (5) nondrug causes of liver injury, (6) previous information on the hepatotoxicity of the drug, and (7) response to rechallenge. The total possible score ranges from −9 to +14, and it could be interpreted as follows: 0 or less, excludes the drug as a cause; 1–2, “unlikely”; 3–5, “possible”; 6–8, “probable”; and greater than 8, “highly probable.”

In our patient, the RUCAM score was +6 (time to onset +1; course +3; risk factors 0; concomitant drugs 0; search for nondrugs causes +1; previous information on the hepatotoxicity of the drug +1; response to rechallenge +0), making “probable” the correlation between ceftriaxone and liver injury. Treatment with antibiotics has been reported as a rare cause of drug-induced liver injury (DILI) (8).

In a recent retrospective study by Barman et al. in the adult population treated with ceftriaxone from January 2019 to December 2019, among the 634 patients included in the study, ceftriaxone was associated with liver injury in 19.7% of cases, especially when used along with other medications that are metabolized in the liver (9).

In the pediatric population, the large, multi-database, population-based, case–control study of Ferrajolo et al. demonstrated that the use of antibiotics is associated with a threefold increased risk of liver injury compared with past use. It underlines the importance of considering these reactions in patients undergoing antibiotic therapy (10).

Mechanisms of DILI are only partially known and could be distinguished in intrinsic and idiosyncratic hepatotoxicity (11).

Intrinsic hepatotoxicity is predictable and occurs when the dosage administered to the patient goes beyond the toxic dose. Conversely, idiosyncratic hepatotoxicity is unpredictable, unexpected, and independent of the dose administered. Usually, antibiotics are responsible for idiosyncratic DILI (6).

According to the European Association for the Study of the Live (EASL) guideline, case definitions for DILI include one of the following thresholds (12):

1. ≥5 ULN elevation in ALT;
2. ≥2 ULN elevation in alkaline phosphatase (ALP) (in the absence of known bone pathology that causes an increase in ALP levels), particularly with accompanying elevations in concentrations in GGT; and
3. ≥3 ULN elevation in ALT and simultaneous elevation of TBL concentration exceeding 2 ULN.

The most common and well-known hepatic adverse effects of ceftriaxone are cholelithiasis and/or biliary sludge (13).

Ceftriaxone-associated cholelithiasis is dose-dependent and usually asymptomatic. However, it may present with abdominal pain, nausea, and emesis. It could be easily diagnosed with an abdominal ultrasound. Generally, this condition resolves over days or months after cessation of therapy (13).

Other possible adverse effects of ceftriaxone could be acute cholecystitis and hematologic anomalies, including eosinophilia, thrombocytosis, leukopenia, thrombocytopenia, and neutropenia (14).

To the best of our knowledge, only six cases of ceftriaxone-induced hepatitis are described (3, 5, 15–18).

Table 2 summarizes the main findings of these cases. Of these, only two cases are described in the pediatric age. In particular, Peker et al. described a case of a 12-year-old boy who developed acute hepatitis after 6 days of treatment with ceftriaxone for acute tonsillitis. Once diagnosed, the patient was treated with methylprednisolone boluses. Normalization of transaminase values and a reduction of gamma-GT values were achieved in 10 weeks (3). Bell et al. described a case of a 17-year-old female with sickle cell anemia who developed ceftriaxone-induced hemolytic anemia and severe hepatitis 4 days after starting the therapy. Unfortunately, this patient subsequently developed multiorgan failure and died (5).

**Table 2:** Ceftriaxone-induced hepatitis in the literature.

Author, year, nation, reference	Patient age, sex	Reason for treatment with ceftriaxone	Onset of hepatitis from the start of ceftriaxone and ceftriaxone dosage	Liver function test (AST, ALT, g-GT, ALP, INR)	Liver biopsy	Treatment for drug-induced hepatitis	Other complications	Outcome
Nadelman, 1991, USA, (16)	43 years, male	Seronegative lyme disease	4 days after stopping a 3-week course of ceftriaxone 2 g/day of ceftriaxone	Not reported	Not performed	None	Sore throat, rigors, fever, granulocytopenia A bone marrow biopsy was consistent with drug-induced granulocytopenia	Discharge
Longo, 1998, France, (15)	80 years, male	Bronchopneumonia	12 days 2 g/day of ceftriaxone	ALT 385 U/L, AST 525 U/L, g-GT 795 U/L, ALP 720 U/L, INR NA	Not performed	Discontinuation of ceftriaxone	Autoimmune hemolytic anemia treated with steroids and immunoglobulins	Discharge
Bell, 2005, USA, (5)	17 years, female with sickle cell anemia	Pneumonia	4 days ceftriaxone dosage not reported	ALT 8098 U/L, AST 21,575 U/L, g-GT NA, ALP NA, INR 2.23	Not performed	Discontinuation of ceftriaxone	Acute hemolytic anemia, renal and multiorgan failure	Death
Peker, 2009, USA, (3)	12 years, male	Tonsillitis	6 days 50 mg/kg/day	ALT 871 U/L, AST 819 U/L, g-GT 285 U/L, ALP 143 U/L, INR NA	Not performed	Discontinuation of ceftriaxone. Pulse methylprednisolone	None	Discharge
Kaur, 2011, India, (17)	24 years, female	Postoperative treatment after open cholecystectomy	3 days 1 g/day	ALT 164 U/L, AST 148 U/L, g-GT NA, ALP 580 U/L, INR NA	Not performed	Discontinuation of ceftriaxone	None	Discharge
Guarino, 2022, Italy, (18)	77 years, female	Bronchopneumonia	2 days 2 g/day	ALT 6111 U/L, AST 11,961 U/L, g-GT 157 U/L, ALP 80 U/L, INR 24.92	Not performed	Discontinuation of ceftriaxone. Pulse methylprednisolone	Prolonged INR treated with vitamin K infusion	Discharge

AST, aspartate aminotransferase; ALT, alanine aminotransferase; Gamma-GT, gamma-glutamyl transferase; INR, international normalized ratio.

The treatment of ceftriaxone-induced hepatitis could include corticosteroid administration (3, 18).

Our patient spontaneously improved without any steroid treatment and did not develop hemolytic anemia, which is another common adverse effect of ceftriaxone (19).

## Conclusion

Ceftriaxone is generally a well-tolerated third-generation cephalosporin widely used in the pediatric age. However, this case describes a rare form of drug-induced cholestatic hepatitis in pediatric settings. It highlights that clinicians should be aware of its possible rare hepatotoxic effect to stop the drug promptly.

## Data Availability

The original contributions presented in the study are included in the article/Supplementary Material, further inquiries can be directed to the corresponding author.
